# Profiles of Loneliness and Ostracism During Adolescence: Consequences, Antecedents, and Protective Factors

**DOI:** 10.1007/s10578-024-01664-8

**Published:** 2024-02-09

**Authors:** Noona Kiuru, Katariina Salmela-Aro, Brett Laursen, Kati Vasalampi, Marguerite Beattie, Mari Tunkkari, Niina Junttila

**Affiliations:** 1 Department of Psychology, University of Jyväskylä, P.O. Box 35, 40014 Jyvaskyla, Finland; 2 University of Helsinki, Helsinki, Finland; 3 Florida Atlantic University, Boca Raton, USA; 4 University of Turku, Turku, Finland; 5 University of Jyväskylä, Jyvaskyla, Finland

**Keywords:** Adolescence, Loneliness, Ostracism, Profiles, Consequences, Antecedents

## Abstract

This longitudinal study (*N* = 1078, 46% boys; 54% girls) examined profiles of loneliness and ostracism during adolescence and their consequences and antecedents. Longitudinal latent profiles analyses identified four distinct profiles: (1) High emotional loneliness (25%), High and increasing social loneliness (15%), High peer exclusion and high social impact (9%) and No peer problems (51%). Subsequent internalizing problems were typical for the High and increasing social loneliness profile and externalizing problems for the High emotional loneliness and High peer exclusion and high social impact profiles. Furthermore, effortful control, prosocial skills, and relationship quality with parents and teachers were highest in the No peer problems profile, whereas the High and increasing social loneliness profile had the lowest self-esteem and was characterized by low surgency/extraversion, high affiliativeness, and high negative affectivity.

## Introduction

All humans have a fundamental need to belong, that is, feeling relatedness to other people (cf. [1, 2]). Anything that threatens meeting this need can be seen as a threat to equality and existence. Unfortunately, the need to belong is not fulfilled for as much as 10% to 20% of adolescents who continuously feel lonely and socially isolated [3, 4]. Loneliness and isolation are particularly painful experiences during adolescence, when individuals have an intensified need to be accepted in their peer group [5, 6]. In this study we examine two interlinking yet unique forms of social outsiderhood, that is, being lonely and being ostracized. *Loneliness*—defined as an unpleasant emotional response arising from the subjective feeling of discrepancy between actual and desired social connections—and *ostracism*—defined as being excluded and/or ignored by other individuals or groups—can have long-term detrimental effects on psychosocial well-being and health while also endangering safety in society [7, 8]. Although loneliness and ostracism are highly intertwined [9], the reasons for loneliness and ostracism may be different, i.e. ostracism is related to others’ action as such that others are ignoring or rejecting you, while loneliness refers to a subjective feeling that your own social needs for belongingness are not met. Loneliness and ostracism have been studied mostly separately, and among adults. Hence, little is known about their combined profiles and related consequences for mental well-being, delinquency and school engagement during adolescence. Knowledge of different antecedents underlying these profiles is also limited. Consequently, this longitudinal study examined the profiles of loneliness and ostracism during adolescence and their consequences, antecedents, and protective factors.

## Profiles of Loneliness and Ostracism During Adolescence

Loneliness arises from the mismatch between the desired and actual social connections [4, 7, 10, 11]. This discrepancy entails a distressing feeling to perceived social isolation and longing for human contact [4, 11]. Loneliness differs from being alone and is not equal to objective social isolation. People can live relatively solitary lives and not feel lonely, and on the contrary, they can have a busy social life and wide social networks and still feel lonely (see also [7, 12]). Loneliness is also a multidimensional phenomenon consisting of the dimensions of social and emotional loneliness [13], see also [14–17]. *Social loneliness* refers to longing for an absent broader social network, whereas *emotional loneliness* refers to longing for an absent intimate and close attachment with a friend or friends [13]. Previous research has also shown that experiences of social and emotional loneliness do not necessarily overlap, for instance, an adolescent may have satisfying broader peer networks but still lack close friends [16, 18–20].

Ostracism, in turn, is defined as being ignored and/or excluded by individuals or groups [8, 21]. *Explicit exclusion or rejection* occurs when the source is explicitly denying the target’s social request. *Ignoring,* in turn, is a more subtle form of exclusion that undermines targets’ sense that other people acknowledge their existence [21, 22]. Being excluded or ignored signals a threat to individuals [8]. Within even a brief episode of ostracism, individuals tend to report distress, anger, sadness, and lower levels of belonging, self-confidence, control, and meaningful existence [23, 24]. In the present study, the explicit exclusion aspect of ostracism was operationalized as peer-rated rejection by the peer group and ignoring the aspect of ostracism was operationalized as peer-rated neglect by the peer group (i.e., low social impact or visibility in the peer group, for a review of related sociometric literature, see also [25]). However, instead of predetermined cutoffs and quantitative indicators with nonoverlapping distributions, our focus was on naturally occurring subgroups [26] of peer exclusion and ignoring by the peer group.

An important aspect of adolescent loneliness and ostracism is the continuity vs. discontinuity of these experiences across time. Although most adolescents experience relatively low levels of social isolation over time, some adolescents experience prolonged loneliness [4, 27–29]. Regarding ostracism, active exclusion or rejection by the peer group has also been found to be a rather stable phenomenon during adolescence [30–32]. However, as far as we know no previous studies have attempted to simultaneously investigate the extent of overlap and profiles of different aspects of loneliness (i.e., social and emotional) and ostracism (i.e., excluding and ignoring). Further understanding of different combinations of loneliness and ostracism and related consequences, antecedents, and protective factors could be used to guide designing interventions to alleviate these experiences [16].

## Psychological Consequences of Prolonged Loneliness and Ostracism

Although loneliness in itself is a negative experience, it serves some adaptive functions according to the evolutionary theory of loneliness [33]. From the evolutionary perspective, it has been proposed that loneliness prepares individuals to cope with a potentially unsafe environment without the protection of others. As a result, loneliness is often accompanied by an increased vigilance for social threats [34]. At the same time, it has been suggested that loneliness also mobilise the so-called re-affiliation motive, which helps individuals to reconnect with significant others and, therefore, reduce their feelings of loneliness. However, not all lonely individuals are able to resolve their feelings of loneliness [27]. Such prolonged loneliness can lead to cognitive overload, deteriorating social functioning, physical and mental health problems, and even suicidality [3, 27, 35]. Similarly, as an insidious form of social violence, ostracism also activates social pain reactions in the brain, and if prolonged, it has adverse well-being consequences, including increased risk for psychiatric disorders, impaired immune functioning and even the risk for violent radicalization [8, 36, 37].

Many prospective studies have shown that stable and high levels of loneliness during adolescence is a risk factor for poor subsequent mental health including elevated levels of internalizing problems, such as depressive symptoms, anxiety and psychosomatic symptoms [4, 29, 38–40]. Prolonged loneliness and ostracism have also been proposed as risk factors for subsequent externalizing problems, such as conduct problems, violent acts, and criminality [3, 28, 41]. Moreover, some studies have shown that loneliness can have detrimental consequences in other domains in life such as education and work. For example, Benner [42] showed that adolescents in the increasing and chronically high loneliness trajectories tend to make less academic progress [42], be at higher risk for school dropout [3] and lower subsequent income and even labor market exclusion [17]. However, little is known about the consequences of different profiles of loneliness and ostracism on adolescent psychosocial well-being and educational outcomes. Thus, in the present longitudinal study we aim to shed further light on these profiles and related consequences.

## Sociodemographic, Individual and Contextual Antecedents of Loneliness and Ostracism

In order to more effectively prevent detrimental consequences of loneliness and ostracism, it is also pivotal to increase understanding of various sociodemographic, individual and contextual risk and protective factors underlying these distinct forms of social outsiderhood (see also [16]). Previous research has linked *sociodemographic* factors, such as socioeconomic status and sex to loneliness. Depending on their socioeconomic status, parents may have differential financial resources and social capital to support their children’s development [43]. The level of education is highly hereditary [44, 45] and it affects not only the quality of the home learning environment, parental action, and investment in resources, but also children’s abilities and skills [46, 47]. Accordingly, results from the few previous studies show that low socioeconomic status is related to higher levels of loneliness among adolescents [17, 28, 48, 49]. The results may indicate that parents with high socioeconomic status may more actively facilitate their children’s participation in different activities and hobbies, which may increase their inclusion in a broader peer network. Previous studies, in turn, have reported mixed results on sex differences in regards to overall loneliness [50, 51]. However, studies using two-dimensional measures of loneliness have consistently found that adolescent boys report higher levels of emotional loneliness than girls do and that adolescent girls experience more social loneliness than boys do [16, 20, 52].

*Temperament,* which refers to constitutionally rooted individual differences in reactivity as well as to the self-regulation processes modulating this reactivity [53–55], may also play an important role in the development of loneliness and ostracism. Temperament may influence social interactions through the individual’s responses to new social encounters, their reactivity and self-regulation during social situations, and recovery in response to a social threat [54, 56]. For example, high levels of negative emotionality, withdrawn behavior, and difficulties to regulate own emotions and behaviors may pose risks to social relationships as they are linked to behavioral deficiencies [57], that may undermine the opportunities to initiate and maintain positive social relationships. In previous research, neuroticism, introversion, and low agreeableness have all been linked to relationship difficulties and poor social integration [3, 58, 59]. However, it is still an open question whether some temperament dimensions are particularly relevant for some specific combinations of adolescent loneliness or ostracism.

Based on the resilience framework [60], it is also be essential to pay attention to *adaptive individual competences* (here prosocial skills, self-esteem; see also [61]) and *contextual protective factors* (here quality of relationships with parents and teachers; see also [62]) that might contribute to avoiding adverse trajectories of loneliness and ostracism during adolescence. Social skills are important in tackling relational challenges, keeping up social relationships, participating in group activities, and being independent and active in social interactions [3, 61, 63, 64]. Self-esteem, in turn, appears as feeling socially competent, valuable and resilient [61, 65, 66]. A recent study by Sakiz et al. [61] showed that both high self-esteem and high social skills protect adolescents against loneliness and ostracism. There are also some previous findings to suggest that high parental affection, closeness, and care could protect against loneliness [27]. Despite increased orientation towards peers during adolescence, parents remain as major providers of social support (see also [62, 67]). However, less is known about teachers’ possible protective role and whether parental and teacher support are particularly relevant for some specific aspects of adolescent loneliness or ostracism.

## Research Questions and Hypotheses

This longitudinal study aimed to provide increased understanding on profiles of adolescent loneliness and ostracism and related antecedents, consequences, and protective factors. More specifically, the following research questions were asked:

(1) What kinds of profiles of loneliness (i.e., social and emotional) and ostracism (i.e., exclusion and ignoring) can be identified during adolescence?

### H1

First, it was expected that the largest subgroup would consist of adolescents experiencing neither loneliness nor ostracism (i.e., a group with no peer problems). Second, based on the theories of multidimensionality of loneliness [13], see also [17] and ostracism [8], it was expected that four different relatively stable profiles of loneliness and ostracism would be identified among adolescents: socially lonely, emotionally lonely, excluded, and neglected adolescents.

(2) How are identified profiles of loneliness and ostracism related to adolescents’ subsequent internalizing and externalizing problems and engagement with upper secondary studies?

### H2a

Adolescents with no peer problems were expected to be best adjusted regarding all well-being and engagement outcomes.

### H2b

Adolescents suffering from stable high loneliness were expected to be at risk for both higher subsequent internalizing and externalizing problems.

### H2c

Adolescents suffering from stable high ostracism were expected to be at risk especially for subsequent externalizing problems and police contact.

(3) How are (a) sociodemographic and ability-related (i.e., sex, fluid intelligence, level of parental education) and personality-related (i.e., temperament) antecedents, individual adaptive competencies (i.e., social skills, self-esteem) and contextual protective factors (i.e., quality of parent and teacher relationships) related to membership in these profiles?

### H3a

A lower level of parental education was expected to be related to increased loneliness and girls were expected to be more vulnerable to social loneliness and boys were expected to be more vulnerable to emotional loneliness.

### H3b

Regarding adolescent temperament dimensions, low surgency/extraversion, high negative affectivity, and low effortful control were expected to be related to profiles characterized by stable high loneliness.

### H3c

Prosocial skills and self-esteem, as well as perceived quality of relationships with parents and teachers, were expected to be higher for adolescents without peer problems, as such that these factors would act as protective factors against stable high profiles of loneliness and ostracism.

## Method

### Participants

Our sample comprises 1078 adolescents (46% boys, *M* = 12.31 years at the outset, *SD* = 0.37) who were drawn from a longitudinal study conducted in Finland from 2014 to 2018. The adolescents were recruited from one large town (about half of the age cohort) and one middle-sized town (whole age cohort) in Central Finland. Both towns included semi-rural areas with smaller schools. Target schools (n = 30) were selected according to their location and size, with the aim of achieving a sufficient sample size and enabling extensive data collections throughout the whole research project. The aim of the larger study was to follow-up students through the transition from primary school to upper secondary education, thus primary schools were selected from areas where all children transfer to particular secondary schools instead of dispersing to different locations. The selection and recruitment of the schools was done in cooperation with the local school authorities.

In all, 97% of the adolescents had Finnish as their heritage language, 1% were bilingual, and 2% of the adolescents spoke a language other than Finnish as their mother tongue. Seventy-five percent of the families were nuclear families, 13% were single-parent families, 12% were blended families, and 1% were other types of families.

A total of 4% of adolescents’ mothers (8% of fathers) were not educated beyond nine years of basic education, 31% of mothers (42% fathers) had completed upper secondary education, 38% of mothers (29% fathers) had a bachelor’s degree, and 27% of mothers (21% of fathers) had a master’s degree or higher. The sample was fairly representative; however, compared to same-age Finnish population, the parents were slightly more educated [68] and single-parent households underrepresented and two-parent households overrepresented [69].

### Procedure

The data were collected in the paper and pencil format by two trained research assistants during normal school hours. Depending on the time point the data collection session at schools lasted from 45 to 90 min (with a 15-min break in the middle). Informed consent was obtained from all the participants of the study. Parental written consent and child assent were required for student participation. Teachers of the participating classrooms also gave their written consent for the data collection to be conducted during the lessons. The procedures of the study follow the principles of the Helsinki Declaration on research with human subjects and the larger longitudinal study was approved by the ethics committee of the local university. Six waves of data were collected: (a) fall of the sixth grade (T1), (b) fall of the seventh grade (T2, c) spring of the seventh grade (T3), (c), fall of ninth grade (T4), d) spring of ninth grade (T5), and e) fall of tenth grade (i.e., upper secondary education (T6). Loneliness and ostracism were measured at the first five time point (T1, T2, T3, T4, and T5), antecedent variables were measured at T1, and consequent variables were measured at T6. A total of 841 (78%) adolescents filled in the questionnaire at T1, 834 (77%) at T2, 825 (76%) at T3, 884 (82%) at T4, 885 (82%) at T5 and 776 (72%) at T6.

### The Finnish Educational System

In Finland, nine years of compulsory basic education is divided into primary school (grades 1–6) and lower secondary school (grades 7–9). In primary and lower secondary school, all students follow the same curriculum and are taught at the same academic level. After completing nine years of basic education, adolescents can choose mainly from two upper secondary education options: an academic track or a vocational track [70]. The academic track (i.e., upper secondary general education) provides general education, but it does not qualify students for any particular occupation. The vocational track (i.e., upper secondary vocational education) includes upper secondary qualifications and provides the basic skills required in the field.

## Measures

### Social and Emotional Loneliness (T1, T2, T3, T4, T5; grades 6–9)

Social and emotional loneliness were assessed at all five timepoints. Adolescents’ *social loneliness* was measured with a widely used single item question, derived from the Health Behaviour in School-Aged Children (HBSC) study [71, 72], ‘*Do you ever feel lonely*?’ responded on four-point scale: 0 = No, 1 = Yes, sometimes, 2 = Yes, quite often, 3 = Yes, very often [4]. This direct measure of loneliness, tapping mainly social loneliness (i.e., closest correlation with the UCLA item ‘I feel being left out by others’ in the Finnish national data) has been widely used in national and international surveys and has been shown to have good face, concurrent, and predictive validity [73–76].

*Emotional loneliness,* in turn, was operationalized as adolescents’ low experienced closeness with their friends [12, 13]. Adolescents were asked to rate their experiences of closeness with their best friends at school (seven items; e.g., “If I have problems I can talk about them with my friend; all αs > 0.80) using the Friendship Qualities Scale [77], see also [62]. The adolescents answered these questions using a five-point Likert scale (1 = not true at all; 5 = completely true). In our analyses, the items were reversed to measure emotional loneliness.

### Exclusion and Ignoring (T1, T2, T3, T4, T5; grades 6–9)

Adolescents completed identical peer nomination surveys at each time point. The adolescents were asked to nominate up to six peers from their school class with whom they most liked to spend time and six peers with whom they least liked to spend time. Sociometric nominations have proved valid, stable, and reliable assessments of adolescents’ status in their peer group [78].

The *peer exclusion* (rejection) score was calculated as the number of received negative (disliked) peer nominations (see also [25, 31, 79]). The *ignoring* aspect of ostracism was operationalized as low peer-rated social impact or visibility in the peer group (neglect by the peer group). The related social impact score was calculated as the sum of number of received positive (liked) and negative (disliked) peer nominations (see also [25]). Both exclusion and social impact scores were divided by school class size -1 in order to eliminate the impact of school class size for the amount of received peer nominations (see also [80]).

### Consequences of Loneliness and Ostracism (T6; Upper Secondary Education)

#### Engagement to Upper Secondary Studies

Adolescents’ disengagement from upper secondary studies was measured with two indicators: school dropout intentions and school absences.

In the first year of upper secondary school adolescents’ *intentions to drop out of school* were measured with two items (e.g., “Have you considered dropping out from your school or field of study?”; see also [81] that were rated on a five-point scale (1 = not at all, 5 = very often, α = 0.78).

Adolescents were also asked to report their *school absences* by answering the following questions (see also Finnish School Health Promote Study 2022 [82]): “How many days have you been absent from school during this semester because of (1) sickness, (2) truancy or skipping school and (3) other reasons (e.g., travelling)”. The answer options were 1 = none, 2 = 1–2 days, 3 = 3–5 days and 4 = over 5 days. Different reasons for school absences were treated as different variables.

#### Internalizing Problems

Adolescents’ internalizing problems were measured with five items (e.g., “I'm usually unhappy, down or weepy”,” I suffer from many fears”) derived from the emotional symptoms subscale of the Strengths and Difficulties Questionnaire (SDQ; [83]) on a three-point scale from 0 to 2 (0 = Not true,2 = Certainly true). A mean score (range of scale 0–2) across the five items was calculated to measure internalizing symptoms (α = 0.71).

#### Externalizing Problems

Adolescents’ externalizing problems were measured with two adolescent-reported indicators: conduct problems and police contact.

*Conduct problems* were measured with eight items (e.g., “I often fight with other adolescents”) derived from the Strengths and Difficulties Questionnaire (SDQ; [83]), see also [84] on a scale from 0 to 2 (0 = Not true,2 = Certainly true). A mean score (range of scale 0–2) across the items was calculated (α = 0.70).

*Police contact* was measured with the item drawn from the Finnish School Health Questionnaire [82]. Adolescents were given the instruction and the following question: “Sometimes adolescents do some things that are forbidden. How often you have been in contact with the police due to your illegal acts?” Adolescents responded on a four-point scale (0 = Never, 1 = Once during the last three years, 2 = 2–3 times during the last three years, 3 = More often than three times during the last 3 years).

#### Using Welfare Services

Adolescents were asked to report on the scale ranging from 0 (hardly never) to 5 (more often than once a week) how often they had used different welfare services: (1) student welfare, (2) welfare offices, (3) psychologists, (4) doctor, (5) psychiatrist, (6) social work, and (7) other welfare services*.* A mean score across the items was calculated to measure the extent of using welfare services (α = 0.72).

### Antecedents of Loneliness and Ostracism (T1; at the End of Primary School)

#### Sex

Adolescent biological sex was coded as 0 = girl and 1 = boy.

#### Fluid Intelligence

Adolescents’ nonverbal intelligence was assessed using the Raven Standard Progressive Matrices [85]. Raven’s test consists of diagrams with one part missing and participants are asked to choose the correct part that would complete each design. In our study, half of the items (i.e., 30) were used and alternating items were presented (see also [86]). A number of the correct answers was calculated (maximum score = 30, α = 0.81).

#### Temperament

To assess adolescent temperament the adolescents filled in the Finnish version of the Early Adolescent Temperament Questionnaire—Revised (EATQ-R; [87–89]); for validity in the Finnish sample, see [90]. The questionnaire consisted of 71 statements on a five-point Likert scale (1 = almost never true, 5 = almost always true) measuring temperamental surgency/extraversion, negative affectivity, effortful control, and affiliativeness. Mean scores for surgency/extraversion (α = 0.73), negative affectivity (α = 0.86), effortful control (α = 0.79), and affiliativeness (α = 0.82) were computed.

#### Prosocial Skills

Adolescents’ prosocial behavior was measured with five items (e.g., “I’m kind to younger people”) from the Strengths and Difficulties Questionnaire (SDQ; [83]) on a scale from 0 to 2 (0 = Not true, 2 = Certainly true). A mean score (range of scale 0–2) across the five items was computed (α = 0.72).

#### Self-esteem

Adolescents’ self-esteem was assessed with an abbreviated version of Rosenberg’s [66] self-esteem scale. The scale consisted of five items (e.g., “On the whole, I am satisfied with myself”), that the adolescents were asked to rate using a scale from 1 (I totally disagree) to 5 (I totally agree). A mean score of the items was calculated (α = 0.73).

#### Closeness to and Conflict with Parents

The adolescents were asked to rate their experiences of closeness (five items; e.g., “I have a close and warm relationship with my mother/father”) and conflict (six items; e.g., “I often argue with my mother/father”) with their mothers (or stepmothers) and fathers (or stepfathers) using the Child Parent Relationship Scale (CPRS; [91]), see also [62]. They answered the questions on a five-point Likert scale (1 = not true at all; 5 = completely true) and the mean scores were calculated across the items to measure the adolescents’ perceived closeness to and conflict with their parents (all αs > 0.70).

#### Closeness to and Conflict with Primary School Class Teacher

The adolescents were also asked to rate their experiences of closeness (five items; e.g., “I have a close and warm relationship with my teacher”) and conflict (six items; e.g., “I often argue with my teacher”) with their classroom teacher using the Student–Teacher Relationship Scale (STRS-Short Form; [92]), see also [62, 93]. They answered the questions on a five-point Likert scale (1 = not true at all; 5 = completely true). The mean scores (all αs > 0.70) were calculated across the items to measure adolescents’ perceived closeness to and conflict with their class teacher.

### Statistical Analyses

Adolescents’ profiles based on loneliness (emotional and social) and ostracism (exclusion and ignoring) were identified using longitudinal latent profiles analyses (LLPA, mixture modeling; [94] [26]); in Mplus (Version 8.9, [95]). LLPA aims to identify the smallest number of homogeneous profile groups that explain the most variation in the observed continuous variables, minimizing within-profile differences on observed indicator variables and maximizing between-profile differences on the same variables. Before LLPA, adolescents’ scores of five repeated measurements of adolescents’ loneliness and ostracism dimensions were standardized according to the mean and standard deviation of the same construct at the first time point in order to make scales of different variables comparable and to also be able to reveal relative increases or decreases in relation to the first time point.

The proportions of missing data for the study variables ranged from 0 to 33% (M = 18%, SD = 11%). Our data were not missing completely at random: Little’s [96] missing-completely-at-random test: χ2(4,789) = 5,872, *p* < 0.001. Hence, the standard missing-at-random (MAR) approach was applied, that is a weaker condition for missingness data than missing completely at random. In the MAR situation, missingness does not depend on the unmeasured variables but can depend on the values of variables included in the analyses [96]. Assuming MAR, missing data were dealt with full information maximum-likelihood (FIML) estimation with robust standard errors [94], allowing adolescents with incomplete data to be included in the models.

We used the following indices to select the appropriate number of latent profiles in the mixture models: (a) Akaike’s information criterion (AIC) and the Bayesian information criterion (BIC) with smaller information criterion values pointing a better model fit; (b) the Lo–Mendell–Rubin adjusted likelihood ratio Test (LMR), and the Vuong–Lo–Mendell–Rubin likelihood ratio test (VLMR) (*p* < 0.05 indicating that the k − 1 group model must be rejected in favor of a model with at least k profiles); and (c) the practical usefulness, theoretical justification, and interpretability of the latent profile solutions (see [97, 98]). Comparison between latent profiles in regard to consequences and antecedents was conducted using the Mplus auxiliary function with the BCH/du3step method [95].

## Results

### Profiles of Loneliness and Ostracism (RQ1)

**Fig. 1:**
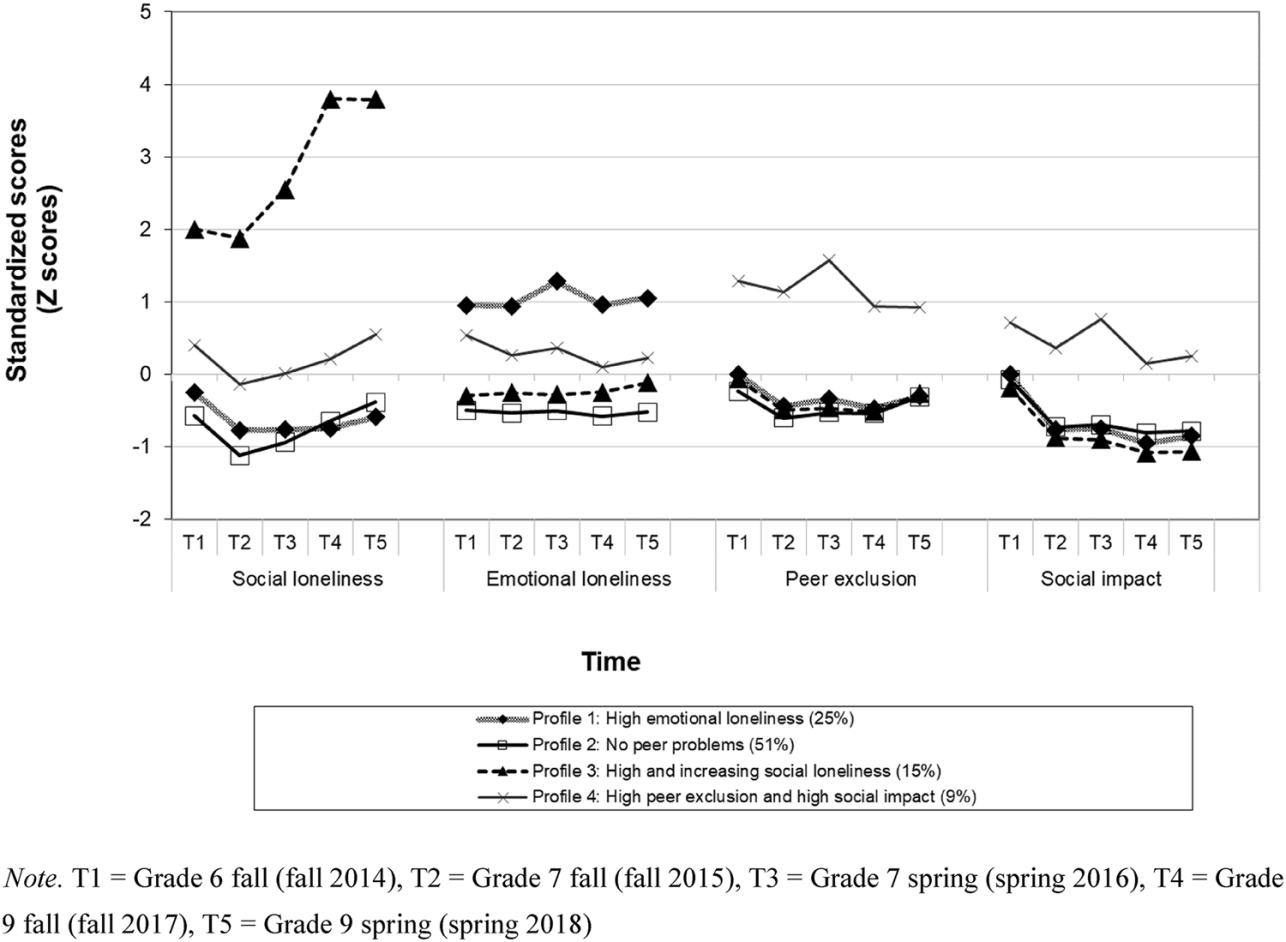
Profiles of loneliness and ostracism. T1—Grade 6 fall (fall 2014), T2—Grade 7 fall (fall 2015), T3—Grade 7 spring (spring 2016), T4—Grade 9 fall (fall 2017), T5—Grade 9 spring (spring 2018)

Descriptives and correlations between loneliness and ostracism variables are shown in Table 1. In turn, the goodness-of-fit indices of the LLPAs for adolescents’ loneliness and ostracism suggested that the four-profile solution fitted the data best (Table 2). The average individual posterior probabilities for being assigned to a specific latent profile in the four-profile model were 0.91, 0.92, 0.87 and 0.97, indicating a clear classification for interpretation of the profiles. Table 3 shows estimated mean scores of latent profiles in the original scale, whereas Fig. 1 illustrates estimated latent mean profiles in the standardized scale. Based on the mean scores and differences between the latent profiles, the loneliness and ostracism subgroups of adolescents in the four-profile solution were labeled as follows: (1) *High emotional loneliness* (25%, *n* = 270), *High and increasing social loneliness* (15%, *n* = 155), *High peer exclusion and high social impact* (9%, *n* = 97) and *No peer problems* (51%, *n* = 556).

**Table 1:** Correlations between loneliness and ostracism variables

Variables	1	2	3	4	5	6	7	8	9	10	11	12	13	14	15	16	17	18	19	*M*	*SD*
1. Social loneliness at T1	–																			1.64	0.73
2. Social loneliness at T2	0.43^a^	–																		1.50	0.66
3. Social loneliness at T3	0.35^a^	0.49^a^	–																	1.56	0.74
4. Social loneliness at T4	0.32^a^	0.37^a^	0.43^a^	–																1.67	0.79
5. Social loneliness at T5	0.25^a^	0.33^a^	0.39^a^	0.52^a^	–															1.72	0.81
6. Emotional loneliness at T1	0.04	0.03	− 0.01	− 0.05	− 0.09^c^	–														1.87	0.72
7. Emotional loneliness at T2	0.02	0.06	− 0.00	− 0.01	− 0.08^c^	0.60^a^	–													1.83	0.71
8. Emotional loneliness at T3	0.01	0.02	0.03	− 0.04	− 0.08^c^	0.56^a^	0.65^a^	–												1.90	0.81
9. Emotional loneliness at T4	0.07	0.01	− 0.03	0.00	− 0.06	0.52^a^	0.56^a^	0.60^a^	–											1.79	0.67
10. Emotional loneliness at T5	0.06	0.01	− 0.03	− 0.02	0.00	0.42^a^	0.45^a^	0.52^a^	0.67^a^	–										1.85	0.74
11. Peer exclusion at T1	0.16^a^	0.13^a^	0.10^b^	0.04	0.03	0.20^a^	0.18^a^	0.15^a^	0.16^a^	0.09^c^	–									0.14	0.15
12. Peer exclusion at T2	0.05	0.11^b^	0.10^c^	0.06	0.08	0.16^a^	0.15^a^	0.16^a^	0.12^b^	0.13^a^	0.44^a^	–								0.08	0.11
13. Peer exclusion at T3	0.06	0.09^c^	0.07	0.04	0.05	0.18^a^	0.14^a^	0.14^a^	0.14^a^	0.14^a^	0.40^a^	0.65^a^	–							0.09	0.12
14. Peer exclusion at T4	0.10^b^	0.09^c^	0.02	0.02	0.02	0.17^a^	0.13^b^	0.10^b^	0.12^b^	0.11^b^	0.37^a^	0.46^a^	0.63^a^	–						0.08	0.10
15. Peer exclusion at T5	0.06	0.05	0.06	0.07	0.06	0.13^b^	0.11^c^	0.06	0.08	0.09^c^	0.28^a^	0.42^a^	0.46^a^	0.52^a^	–					0.12	0.10
16. Social impact at T1	0.01	0.00	0.03	− 0.01	− 0.04	0.09^c^	0.04	0.07	0.06	0.01	0.64^a^	0.18^a^	0.14^a^	0.11^b^	0.13^b^	–				0.38	0.15
17. Social impact T2	− 0.07	− 0.06	0.04	− 0.04	0.04	0.04	− 0.03	0.03	0.00	0.03	0.12^b^	0.59^a^	0.33^a^	0.19^a^	0.07	0.09^b^	–			0.27	0.14
18. Social impact T3	− 0.02	− 0.04	0.01	− 0.04	− 0.01	0.04	− 0.02	0.01	0.02	0.05	0.12^b^	0.34^a^	0.66^a^	0.33^a^	0.10^c^	0.07	0.51^a^	–		0.28	0.14
19. Social impact T4	− 0.02	− 0.02	− 0.05	− 0.07	− 0.05	0.02	− 0.03	0.00	− 0.03	0.01	0.09^c^	0.12^b^	0.23^a^	0.51^a^	− 0.03	0.06	0.28^a^	0.39^a^	–	0.25	0.14
20. Social impact T5	− 0.03	− 0.06	0.04	− 0.01	− 0.07	0.03	0.00	− 0.04	0.01	0.01	0.09^c^	0.17^a^	0.21^a^	0.16^b^	0.70^a^	0.15^b^	0.06	0.09	0.63^a^	0.27	0.11

Range of scale in social loneliness 1–4, Range of scale in emotional loneliness 1–5, range of scale in peer exclusion (rejection) 0–1(proportion scores)), range of scale in social impact (ignoring) 0–1 (proportion scores)

^a^*p* < 0.001

^b^*p* < 0.01

^c^*p* < 0.05

**Table 2:** Fit indices and class frequencies for different numbers of latent profiles

No. of groups	AIC	BIC	aBIC	*p* value of LMR	*p* value of VLMR
1 (*N* = 1078)	54464.21	54663.52	54536.47		
2 (*n*1 = 959, *n*2 = 119)	52117.87	52421.82	52228.07	0.0027	0.0027
3 (*n*1 = 682, *n*2 = 296, *n*3 = 100)	50841.59	51250.19	50989.74	< 0.001	< 0.001
4 (*n*1 = 556, *n*2 = 270, *n*3 = 155, *n*4 = 97)	50287.82	50801.05	50473.90	0.0163	0.0171
5 (*n*1 = 504, *n*2 = 261, *n*3 = 139, *n*4 = 116, *n*5 = 58)	49857.66	50475.53	50081.68	0.4991	0.5025

*AIC* Akaike information criterion, *BIC* Bayesian information criterion, *LMR* Lo-Mendell-Rubin adjusted likelihood ratio test, *VLMR* Vuong-Lo-Mendell-Rubin likelihood ratio test

**Table 3:** Estimated mean scores of and differences between latent profiles in the original scale in regard to loneliness and ostracism variables

Profiles	Profile 1: High emotional loneliness (25%, *n* = 270)	Profile 2: High and increasing social loneliness (14%, *n* = 155)	Profile 3: High peer exclusion and high social impact (9%, *n* = 97)	Profile 4: No peer problems (52%, *n* = 556)		
	M (S.E.)	M (S.E.)	M (S.E.)	M (S.E.)	Overall test *χ*^2^(d*f* = 3)	Pairwise comparisons
Social loneliness at T1	1.57 (0.05)	2.47 (0.09)	1.75 (0.09)	1.42 (0.03)	89.98***	2 > 3 > 1, 4
Social loneliness at T2	1.40 (0.04)	2.36 (0.08)	1.61 (0.09)	1.24 (0.03)	165.75***	2 > 3 > 1 > 4
Social loneliness at T3	1.39 (0.05)	2.61 (0.09)	1.64 (0.10)	1.30 (0.03)	186.16***	2 > 3 > 1, 4
Social loneliness at T4	1.36 (0.04)	3.08 (0.09)	1.72 (0.10)	1.38 (0.03)	315.02***	2 > 3 > 1, 4
Social loneliness at T5	1.43 (0.05)	3.08 (0.09)	1.82 (0.11)	1.46 (0.03)	286.10***	2 > 3 > 1, 4
Emotional loneliness at T1	2.65 (0.05)	1.65 (0.06)	2.25 (0.11)	1.45 (0.02)	523.69***	1 > 3 > 2 > 4
Emotional loneliness at T2	2.64 (0.05)	1.67 (0.06)	2.07 (0.10)	1.43 (0.02)	504.22***	1 > 3 > 2 > 4
Emotional loneliness at T3	2.91 (0.06)	1.68 (0.06)	2.14 (0.12)	1.44 (0.03)	545.04***	1 > 3 > 2 > 4
Emotional loneliness at T4	2.69 (0.05)	1.70 (0.06)	1.94 (0.09)	1.39 (0.02)	694.18***	1 > 3 > 2 > 4
Emotional loneliness at T5	2.77 (0.05)	1.81 (0.07)	2.02 (0.09)	1.44 (0.02)	576.87***	1 > 3 > 2 > 4
Peer exclusion at T1	0.15 (0.01)	0.13 (0.01)	0.34 (0.02)	0.10 (0.01)	116.33***	3 > 1,2,4; 1 > 4
Peer exclusion at T2	0.08 (0.01)	0.07 (0.01)	0.31 (0.02)	0.05 (0.01)	248.70***	3 > 1,2,4; 1 > 4
Peer exclusion at T3	0.09 (0.01)	0.07 (0.01)	0.38 (0.02)	0.06 (0.01)	442.05***	3 > 1,2,4: 1 > 2,4
Peer exclusion at T4	0.07 (0.01)	0.06 (0.01)	0.28 (0.02)	0.06 (0.01)	205.85***	3 > 1,2,4
Peer exclusion at T5	0.10 (0.01)	0.10 (0.01)	0.28 (0.01)	0.09 (0.01)	184.33***	3 > 1,2,4
Social impact at T1	0.38 (0.01)	0.35 (0.02)	0.49 (0.02)	0.36 (0.01)	38.10***	3 > 1,2,4
Social impact at T2	0.26 (0.01)	0.24 (0.01)	0.44 (0.02)	0.26 (0.01)	126.77***	3 > 1,2,4
Social impact at T3	0.26 (0.01)	0.23 (0.01)	0.50 (0.02)	0.27 (0.01)	227.31***	3 > 1,2,4; 4 > 2
Social impact at T4	0.23 (0.01)	0.20 (0.01)	0.40 (0.02)	0.26 (0.01)	101.02***	3 > 1,2,4; 4 > 1,2
Social impact at T5	0.24 (0.01)	0.21 (0.01)	0.42 (0.02)	0.26 (0.01)	133.60***	3 > 1,2,4; 4 > 2

Range of scale in social loneliness 1–4, range of scale in emotional loneliness 1–5, range of scale in peer exclusion (rejection) 0–1(proportion scores)), range of scale in social impact (ignoring) 0–1 (proportion scores)

T1 = Grade 6 fall (fall 2014), T2 = Grade 7 fall (fall 2015), T3 = Grade 7 spring (spring 2016), T4 = Grade 9 fall (fall 2017), T5 = Grade 9 spring (spring 2018)

*p < 0.05

**p < 0.01

***p < 0.001

### Consequences of Profiles of Loneliness and Ostracism (RQ2)

Means, standard errors and differences between the profiles in regard to their subsequent engagement with upper secondary studies and well-being outcomes measured in the first year of upper secondary education are shown in Table 4.

**Table 4:** Estimated means and standard errors and the results of comparisons between latent profiles in regard to consequences measured in the first year of upper secondary school

Variable	Profile 1: High emotional loneliness (25%, *n* = 270)		Profile 2: High and increasing social loneliness (14%, *n* = 155)		Profile 3: High peer exclusion and high social impact (9%, *n* = 97)		Profile 4: No peer problems (53%, *n* = 556)			
	M	SE	M	SE	M	SE	M	SE	Overall test *χ*^2^(d*f* = 3)	Pairwise comparisons
School dropout intentions	1.89	0.08	1.93	0.11	2.08	0.15	1.66	0.05	11.63**	4 < 1,2,3
School absences due to sickness	1.84	0.08	1.79	0.11	2.15	0.15	1.68	0.05	10.44*	3 > 2,4
School absences due to truancy	1.31	0.07	1.14	0.05	1.52	0.14	1.13	0.02	15.15**	3,1 > 2,4
School absences due to other reasons	1.24	0.04	1.44	0.09	1.69	0.12	1.44	0.04	19.44***	3 > 2,4; 2,3,4 > 1
Police contact	0.43	0.07	0.18	0.05	0.65	0.14	0.18	0.03	22.30***	3 > 2,4; 1 > 2,4
Internalizing problems	0.49	0.04	1.15	0.07	0.73	0.07	0.56	0.03	57.83***	2 > 1,3,4; 3 > 1,4
Externalizing problems	0.37	0.04	0.32	0.04	0.42	0.06	0.21	0.02	29.06***	4 < 1,2,3
Using welfare services	0.19	0.05	0.36	0.08	0.28	0.10	0.17	0.02	5.69	2 > 4

The results for *subsequent engagement with upper secondary studies* showed that adolescents in the *No peer problems* profile reported less frequent school dropout intentions than did adolescents in the other profiles. Adolescents in the No peer problems profile had also fewer school absences than adolescents in the other profiles. School absences due to sickness and truancy were more frequent for adolescents in the *High peer exclusion and high social impact* profile than adolescents in the other profiles.

The results for *subsequent well-being outcomes* showed that adolescents in the *High and increasing social loneliness* profile have a higher level of subsequent internalizing problems than adolescents in any other profile. Adolescents in the *High peer exclusion and high social impact* profile also had more internalizing problems than did adolescents in the *No peer problems* or *High emotional loneliness* profiles. The results showed further that adolescents in the *No peer problems* profile had fewer subsequent externalizing problems than did adolescents in any other profiles. In turn, adolescents in the *High peer exclusion and high social impact* and *High emotional loneliness* profiles were more likely to have police contact than adolescents in the *No peer problems* or in the *High and increasing social loneliness* profile. In regard to the extent of using welfare services, the overall test showed no differences between groups. However, pairwise comparisons tentatively showed that adolescents in the *High and increasing social loneliness* profile reported using more welfare services than did adolescents in the *No peer problems* profile.

### Antecedents of Combined Profiles of Loneliness and Ostracism (RQ3)

Means, standard errors and differences between the profiles in regard to individual and contextual antecedents measured in Grade 6 are shown in Table 5.

**Table 5:** Estimated means and standard errors and the results of comparisons between latent profiles in regard to antecedent factors measured in Grade 6

Variable	Profile 1: High emotional loneliness (25%, *n* = 270)		Profile 2: High and increasing social loneliness (14%, *n* = 155)		Profile 3: High peer exclusion and high social impact (9%, *n* = 97)		Profile 4: No peer problems (52%, *n* = 556)			
	*M*	*SE*	*M*	*SE*	*M*	*SE*	*M*	*SE*	Overall test *χ*^*2*^*(df* = *3)*	Pairwise comparisons
Individual antecedents										
Sex (0 = girl, 1 = boy)	0.94	0.02	0.14	0.04	0.71	0.05	0.27	0.02	626.76***	1 > 3 > 4 > 2
Fluid intelligence	22.92	0.31	20.72	0.53	21.73	0.52	23.31	0.19	24.09***	1,4 > 2,3
Surgency/extraversion	3.38	0.04	3.05	0.08	3.52	0.09	3.42	0.04	21.53***	2 < 1,3,4
Affiliativeness	3.06	0.05	3.63	0.06	3.23	0.08	3.54	0.03	90.34***	2,4 > 1,3
Negative affectivity	2.55	0.04	3.11	0.05	2.57	0.07	2.72	0.03	76.38***	2 > 1,3,4; 4 > 1,3
Effortful control	3.45	0.05	3.45	0.05	3.49	0.07	3.65	0.03	21.44***	4 > 1,2,3
Prosocial skills	1.33	0.03	1.51	0.04	1.43	0.04	1.61	0.02	60.34***	4 > 1,2,3; 2 > 1
Self-esteem	3.69	0.05	3.06	0.08	3.59	0.09	3.76	0.04	61.26***	2 < 1,3,4
Contextual antecedents										
Level of mother’s education	4.22	0.10	4.11	0.15	3.98	0.16	4.53	0.07	13.87**	4 > 1,2,3
Level of father’s education	3.86	0.12	4.02	0.17	3.90	0.21	4.00	0.09	0.79	
Closeness with parents	3.59	0.07	3.65	0.10	3.92	0.11	4.24	0.04	64.01**	4 > 1,2,3; 3 > 1
Conflict with parents	2.04	0.05	2.41	0.09	2.18	0.10	1.89	0.04	27.10***	4 < 1,2,3;2 > 1
Closeness with teachers	2.02	0.06	2.13	0.10	2.35	0.11	2.49	0.05	32.80***	4 > 1,2; 3 > 1
Conflict with teachers	1.88	0.09	1.92	0.13	1.79	0.10	1.36	0.04	58.32***	4 < 1,2,3

*p < 0.05

*p < 0.01

***p < 0.001

#### *Individual Antecedents (*Table 5*)*

The results for *sex* showed that girls were overrepresented in the *High and increasing social loneliness* profile and boys were overrepresented in the *High emotional loneliness* and *High peer exclusion and high social impact* profiles.

The results for *fluid intelligence* showed that adolescents in the *No peer problems* and in the *High emotional loneliness* profiles had higher fluid intelligence than did adolescents in the other two profiles.

The results for *temperament* showed that adolescents in the *High and increasing social loneliness* profile had lower surgency/extraversion and higher negative affectivity than adolescents in the other profiles. In addition, adolescents in the *High and increasing social loneliness* and in the *No peer problems* profile had higher affiliativeness than did the adolescents in the *High emotional loneliness* and *High peer exclusion and high social impact* profiles. Adolescents in the *No peer problems* profile also had higher negative affectivity than did adolescents in the *High emotional loneliness* and *High peer exclusion and high social impact* profiles; however, at the same time adolescents with *No peer problems* had higher effortful control than did adolescents in the other profile.

The results for *prosocial skills* showed that adolescents with *No peer problems* had higher prosocial skills than did adolescents in the other profiles. In addition, adolescents in the *High and increasing social loneliness* profile had higher prosocial skills than adolescents in the *High emotional loneliness* profile did.

The results for s*elf-esteem* showed that adolescents in the *High and increasing social loneliness* profile had lower self-esteem than adolescents in the other profiles did.

#### *Contextual Antecedents (*Table 5*)*

The results for the*** l****evel of parental education* showed that mother’s education was higher in the *No peer problems* profile compared to the other profiles. No differences between profiles were found in the level of father’s education.

The results for *quality of parent-adolescent relationships* showed that adolescents in the *No peer problems* profile reported higher closeness and less conflicts with their parents than adolescents in the other profiles did. In addition, adolescents in the *High peer exclusion and high social impact* profile reported higher closeness with their parents than did adolescents in the *High emotional loneliness* profile. Adolescents in the *High and increasing social loneliness* profile also reported more conflicts with their parents than adolescents in the *High emotional loneliness* profile did.

Finally, the results for the *quality of teacher-adolescent relationship* showed that adolescents in the *No peer problems* profile experienced less conflicts with their teacher than adolescents in the other profiles. Adolescents with *No peer problems* also experienced higher closeness with their teacher than adolescents in the *High emotional loneliness* and *High and increasing social loneliness* profiles. In addition, adolescents in the *High peer exclusion and high social impact* profile reported higher closeness with their teacher than did adolescents in the *High emotional loneliness* profile.

## Discussion

This longitudinal study examined the profiles of adolescents’ loneliness and ostracism and their consequences, antecedents and protective factors. Four distinct developmental profiles were identified, which were differentially associated with subsequent internalizing and externalizing problems and engagement with upper secondary education: (1) *High emotional loneliness* (25%), *High and increasing social loneliness* (15%), *High peer exclusion and high social impact* (9%), and *No peer problems* (51%). These developmental profiles also differed in regards to sociodemographic factors, temperament, and individual and contextual protective factors. The results provide new knowledge about different combinations of loneliness and ostracism among adolescents and related antecedents and outcomes. This knowledge can be used to develop procedures to identify subgroups of adolescents at-risk for chronically elevated levels of loneliness and ostracism as well as to design targeted interventions to reduce adolescents’ social outsiderhood.

## Profiles of Loneliness and Ostracism

The results regarding the identified profiles of adolescent loneliness and ostracism were partly in line with our Hypothesis 1. First, as expected (see also [27–29, 99]) the largest subgroup (51%), labelled as *No peer problems*, consisted of adolescents experiencing neither loneliness nor ostracism. The prevalence of this subgroup resembles previous studies among adolescents in which the prevalence of the *No peer problems* group has varied above and below 50% [27–29, 99, 100].

As expected, based on the theory of multidimensionality of loneliness [13], see also [14–17], two distinct subgroups of adolescents suffering from stable high loneliness were also found: *High emotional loneliness* (25%) and *High and increasing social loneliness* (15%). These subgroups of adolescents, consisting altogether of 40% the sample, internally suffered from either emotional or social loneliness that were not combined with more active peer exclusion or peer rejection. These findings support some previous findings suggesting that experiences of social and emotional loneliness do not always overlap [12, 16, 18]. The results also highlight a need to assess both aspects of loneliness in order to reveal actual frequency of loneliness experiences among adolescents.

In contrast to our expectations, two subgroups of actively excluded and neglected adolescents based on different types of ostracism, were not found. Instead, we found one mixed ostracism subgroup of adolescents labelled as *High peer exclusion and high social impact* (9%). This highly stable and distinguishable subgroup of adolescents was characterized by simultaneously high peer rejection and high visibility in the peer group [101, 102]. One possible explanation for the found mixed ostracism profile is that in our study ostracism was measured with peer ratings. Previous research on ostracism among adolescents is scarce and has mainly been conducted either in laboratory settings [103] or with self-report questionnaires [61]. It is possible that ostracism is partly differentially perceived from the perspective of the target of ostracism and from the perspective of the peer group. Another possible explanation for the lacking neglected subgroup is statistical as exclusion and social impact scores were moderately highly correlated (especially within the same measurement points, see Table 1). Separate profiles for exclusion and neglect would be more likely to emerge when the correlation between the two constructs is low.

## Psychological Consequences of Profiles of Loneliness and Ostracism

The results further showed that the psychological consequences of the different profiles of loneliness and ostracism were partly differential. First, in line with our expectations (H2a; see also [29]), adolescents who had no peer problems were best adjusted in all the measured indicators. They had fewer internalizing and externalizing problems and fewer school absences in upper secondary education than did adolescents in the three other profiles, which were characterized by continuous loneliness and ostracism. They also less frequently reported school dropout intentions than adolescents in the other profiles did (H2a).

Second, supporting our expectations (H2b; see also [3, 4, 29, 38]), adolescents in the *High and increasing social loneliness* profile had a higher level of subsequent internalizing problems than did adolescents in any other profile. One possible explanation for these findings is that prolonged feelings of social loneliness (i.e., longing for an absent broader social network) leads to higher stress levels in everyday life, cognitive overload, and hinders social functioning which, in turn, has detrimental consequences on adolescents’ mental well-being [3, 27, 35]. Moreover, adolescents in the *High emotional loneliness* profile, characterized by longing for an absent intimate, close and emotional attachment with a friend or friends, were more likely to have police contact than were adolescents with no peer problems or who suffered primarily from social loneliness. These results support partially the link between chronic loneliness and subsequent delinquency (H2b; see also [3, 28]).

Third, in line with our expectations (H2c; [8]), our results showed that norm-breaking behavior and delinquency were typical consequences of stable high ostracism. School absences due to sickness and truancy were particularly typical for adolescents in the *High peer exclusion and high social impact* profile. Adolescents in the *High peer exclusion and social impact* profile were also more likely to have police contact than adolescents in the other profiles were. Our results support previous studies that have suggested chronic ostracism to be a risk factor for antisocial behavior, radicalization, and violent acts (e.g., [8, 36, 37]). Conduct problems of adolescents, in turn, were not specific for any loneliness or ostracism subgroup, but their level was higher for all the loneliness and ostracism profiles compared to adolescents with no peer problems.

## Antecedents of Profiles of Loneliness and Ostracism

In order to more effectively prevent the adverse consequences of loneliness and ostracism, it is also pivotal to increase understanding of various sociodemographic, individual and contextual risk and protective factors underlying these distinct forms of social outsiderhood.

### The Role of Sociodemographic Factors

In line with our expectations (see also [28, 48, 49]), the sociodemographic and ability-related factors were connected to profiles of loneliness and ostracism. Essentially, the level of mother’s education was higher for adolescents in the *No peer problems* profile compared to the other profiles (H3a) and fluid intelligence was higher for adolescents in the *No peer problems* and in the *High emotional loneliness* profiles than it was among adolescents in the two other profiles. One possible explanation for these findings is that capable adolescents from wealthier homes have better financial opportunities to participate in organized activities that facilitate social interactions with peers than adolescents from poorer families. Parents with high socioeconomic status may to a larger degree facilitate their children’s participation in activities, which may increase their social inclusion in a broader network (see also [17]).

Furthermore, in line with our expectations (H3a; see also [12, 16, 52]), girls were overrepresented in the *High and increasing social loneliness* and boys in the *High emotional loneliness* profile. These results are in line with some previous findings that have shown that compared to boys, girls tend to report having closer and more intimate peer relationships, emphasizing self-disclosure [104], whereas boys tend more often to interact in larger peer group activities [12, 105]. In addition, boys were overrepresented in the *High peer exclusion and high social impact* profile while for adolescents with *No peer problems* the sex distribution was equal. Overall, our results suggest that girls and boys are equally vulnerable for loneliness and ostracism, but they are partly prone to experience different types of social outsiderhood.

### The Role of Adolescent Temperament

Interestingly, regarding the reactive aspects of temperament the results showed that adolescents in the *High and increasing social loneliness* had lower surgency/extraversion and higher negative affectivity than adolescents in all the other profiles did (see also H3b; [29, 106–108]). At the same time, adolescents with *High and increasing social loneliness* had equally high levels of affiliativeness with adolescents with *No peer problems*. Affiliativeness involves concern for others and the desire for closeness with others (independent of extraversion or shyness) [53, 109] and is especially important during adolescence [53, 88, 90]. Our results suggest that high affiliativeness combined with low extraversion and high negative affectivity might increase adolescents’ vulnerability for prolonged social loneliness, experienced as dissatisfaction with current social relationships and longing for a broader social network. Inhibited adolescents who are prone to negative emotionality may find it particularly challenging to form and maintain social relationships and networks, although they attribute high importance to peer relationships.

Furthermore, adolescents in the *Emotionally lonely* and *High peer exclusion and high social impact* profiles, in turn, had lower affiliativeness than did adolescents in the two other profiles and even lower negative affectivity than adolescents with *No peer problems* did. These characteristics may partly protect these adolescents from deeper psychological suffering, internalizing problems and detrimental mental health consequences of social outsiderhood. In turn, regarding the regulative aspects of temperament the results showed that adolescents with no peer problems had higher effortful control than did adolescents in all the other profiles. These findings suggest that high effortful control, which is related to better emotion and behavioral regulation may help adolescents to cope with feelings of loneliness and challenges related to adolescent age (see also [54]).

### Adaptive Individual Competences, and Contextual Protective Factors

The results regarding *individual adaptive competences* showed, first, partly in line with our expectations (H3c), that adolescents with *No peer problems* had higher prosocial skills than adolescents in the other profiles [61]. Thus, high prosocial skills seemed to protect adolescents from ending up with chronic loneliness or ostracism profiles. Accordingly, previous studies have shown that social skills are important in coping interpersonal challenges, maintaining positive social relationships, and participating in community and group activities [3, 61, 63, 64]. When adolescents feel competent in interacting with peers and maintaining positive relationships with other people, they are more likely to initiate and maintain constructive relationships with their social environment (see also [61]). This, in turn, may lead to feelings of belongingness, togetherness and inclusion [110] while decreasing the risk of being ostracized and lonely. Other previous research concerns the link between loneliness and deficits in social skills [3]. It is possible that lonely people exhibit behavior that deters social interaction, such as inappropriate behavior or social anxiety. Hence, training social skills might be one way out from loneliness.

However, our results regarding the protective role of self-esteem were less clear. Although adolescents with *High and increasing social loneliness* had higher prosocial skills than *Emotionally lonely* adolescents, adolescents with *High and increasing social loneliness* had lower self-esteem than adolescents in the other profiles did. These results suggest that even moderate prosocial skills may not be sufficient to protect from social loneliness if one’s self-esteem is low. Self-esteem manifests itself as feeling socially competent, valuable and resilient [61, 65, 66]; and low self-esteem may hinder successful peer interactions. It has been suggested that low self-esteem might foster internal attributions that give rise to a learned helplessness about loneliness [61]. Individuals with low self-esteem are apt to interpret isolation negatively as evidence that confirms their perceptions of social undesirability, and consequent rejection by the group and rejection sensitivity exacerbates this problem (see also [21]). The results of this study were nevertheless correlational: future studies are needed to examine the developmental dynamics and directions of associations between self-esteem and loneliness in greater depth.

Finally, the results regarding *contextual protective factors* were in line with our expectations (H3c; see also [27]) that adolescents in the *No peer problems* profile reported higher closeness and fewer conflicts with their parents and teachers than did adolescents in the other profiles. Thus, high levels of support from parents and teachers seemed to protect adolescents from ending up in the stable high loneliness or ostracism profiles. Warm and trustful relationships with parents and teachers may promote adolescents’ sense of relatedness and, thus, may decrease loneliness and ostracism [111, 112].

## Limitations and Future Directions

Our study has several limitations. First, as part of a broader longitudinal study only widely used single-item direct measure for social loneliness was available (see also [4, 71, 72]). This direct measure of loneliness, tapping mainly social loneliness (i.e., closest correlation with the UCLA item ‘I feel being left out of others’ in the Finnish national data), has been widely used in national and international surveys and has been shown to have good face, concurrent, and predictive validity [40, 74, 75]. The gender differences in our single-item measure also supported previous research by showing that adolescent girls experience more social loneliness than boys do [12, 16, 20, 52]. At the same time, we admit that previous research on associations of different direct and indirect single-item measures of loneliness with different broader scales of emotional and social loneliness is still inconclusive with some other studies also showing closer associations with emotional loneliness [17]. Hence, there is an evident need for future studies that should replicate our results for developmental profiles of emotional and social loneliness with longer and more comprehensive multi-item measures, such as the Relational Provisions Loneliness Questionnaire (RPLQ [19, 20]), that would allow in more depth measurement of different dimensions of loneliness. Similarly, there is a need for future studies about cultural interpretations of the word “loneliness” in order to shed further light on possible cultural differences in the interpretations of the loneliness items. Second, in our study ostracism was measured using peer ratings. It is possible that ostracism is partly differentially perceived from the perspective of the target of ostracism and from the perspective of the peer group. In future studies it would be useful to conduct multimethod studies to examine ostracism in more depth with different complementary measures. Also, future studies with more frequent measurement points and longer timespans during adolescence could potentially shed more light on possible nonlinear patterns and developmental profiles of adolescent loneliness and ostracism.

Third, Finland unfortunately does not have systematic official records of school absenteeism; these registries are still under construction. In our study, we measured school absences and police contact using widely used items in the National and International School Health survey. In the future studies, it would be worthwhile to complement self-reported information with information from official records. Third, the investigated associations with antecedents and consequences are correlational, which precludes making causal conclusions about the associations. Future studies are needed to examine the reciprocal dynamics and the direction of associations between loneliness and ostracism and related antecedents and consequences. Finally, our sample was a homogeneous and a resource-rich sample (as indicated by the high level of educational attainment among the mothers of the participants) consisting of mainly Finnish speaking adolescents. Future studies are needed to examine loneliness and ostracism in more diverse samples and with different immigrant groups.

## Summary

From the scientific, clinical and public health points of view, it is important to identify predictors and outcomes of specific subgroups at risk, such as adolescents who suffer from chronically elevated levels of loneliness and ostracism. Loneliness and isolation are particularly painful experiences during adolescence, when individuals have a heightened need to be accepted in their peer group [5, 6]. Hence, adolescence is a crucial period for reducing loneliness and preventing negative health outcomes and social exclusion [113]. Our study revealed four distinct profiles of loneliness and ostracism, which were differentially associated with subsequent internalizing and externalizing problems and engagement in upper secondary education: (1) *High emotional loneliness* (25%), *High and increasing social loneliness* (15%), *High peer exclusion and high social impact* (9%,) and *No peer problems* (51%). Despite these two forms of social outsiderhood interlinked at separate time points, the developmental pathways seemed to form more nuanced profiles of these types of outsiderhood. For some adolescents, the problems were mostly related to being ostracized by others while the other adolescents had more subjective feeling of being socially or emotionally lonely. The found profiles also meaningfully differed in regards to sociodemographic factors, temperament, and individual and contextual protective factors. The new knowledge on the distinct subtypes of loneliness and ostracism, their consequences and related risk and protective factors can be used to help guide focused interventions to alleviate loneliness and ostracism (see also [16, 114]).

## Data Availability

The datasets generated and/or analyzed during the current study are not publicly available due to ethical restrictions but are available from the corresponding author on reasonable request.

## Appendix: Key Mplus syntaxes

1. Final four class LPA
2. Final four class LPA with BCH to test difference in consequences
3. Final four class LPA to test differences in antecedents
